# Beyond Anthropocentrism: The Moral and Strategic Philosophy behind Russell and Burch’s 3Rs in Animal Experimentation

**DOI:** 10.1007/s11948-024-00504-1

**Published:** 2024-09-11

**Authors:** Nico Dario Müller

**Affiliations:** University of Basel, Philosophical Seminar, Steinengraben 5, Basel, 4051 Switzerland

**Keywords:** 3Rs, Three Rs, Animal research, Animal experimentation, Anthropocentrism

## Abstract

The 3Rs framework in animal experimentation– “replace, reduce, refine” – has been alleged to be expressive of anthropocentrism, the view that only humans are directly morally relevant. After all, the 3Rs safeguard animal welfare only as far as given human research objectives permit, effectively prioritizing human use interests over animal interests. This article acknowledges this prioritization, but argues that the characterization as anthropocentric is inaccurate. In fact, the 3Rs prioritize research purposes even more strongly than an ethical anthropocentrist would. Drawing on the writings of Universities Federation for Animal Welfare (UFAW) founder Charles W. Hume, who employed Russell and Burch, it is argued that the 3Rs originally arose from an animal-centered ethic which was however restricted by an organizational strategy aiming at the voluntary cooperation of animal researchers. Research purposes thus had to be accepted as given. While this explains why the 3Rs focus narrowly on humane method selection, not on encouraging animal-free question selection in the first place, it suggests that governments should (also) focus on the latter if they recognize animals as deserving protection for their own sake.

## Introduction

The 3Rs in animal experimentation– “replace, reduce, refine”– are commonly understood to be a moral principle or a scientific principle whose application is morally desirable (Hobson-West, 2009). But what kind of moral philosophy underpins the 3Rs, if any? Readers of the classic text on the 3Rs, *The Principles of Humane Experimental Technique* (Russell & Burch, 1959), have been perplexed by this question because the authors did not engage in any moral argumentation at all. It is clear enough that Russell and Burch considered animal distress to be of moral concern, but why they thought this and in what terms they thought about it remains obscure. Today, this obscurity still gives rise to conflicting interpretations.

An essential point of contention is whether the 3Rs rest on an *anthropocentric* moral perspective. Anthropocentrism is the philosophical view that humans alone matter morally for their own sake, while anything else, including animals, matters only indirectly insofar as it affects humans (Fox, 1998). Such views are controversial among animal ethicists because they tend not to give animals sufficient moral consideration. The question, in other words, is whether the concept of the 3Rs addresses animal welfare issues in science in a characteristically anthropocentric way. Some scholars argue that anthropocentrism is a key feature of the 3Rs, particularly because they prioritize human research interests over the interests of animals (Vorstenbosch, 2005; Lauwereyns, 2018).^1^ Other commentators claim the exact opposite, that the 3Rs should be understood as non-anthropocentric given their focus on animal suffering in itself rather than any indirect effects on humans (Camenzind & Eggel, 2022), or that they rest on the specific non-anthropocentric approach of utilitarianism (Schuppli et al., 2004; Landi et al., 2015; Walker & Eggel, 2020).

Understanding the values that originally underpinned the 3Rs is still important today because these values shape how the framework operates– what questions it is built to ask and what goals it is built to achieve. The 3Rs are an intellectual and regulatory tool, and it is relevant to know whether that tool was built for a purpose other than the one we are using it for. Numerous countries today allocate significant public resources to 3Rs programs (Neuhaus et al., 2022). This includes jurisdictions in which animals are recognized as beings worth protecting for their own sake (see Kotzmann, 2023). If the 3Rs are indeed rooted in anthropocentric values, thus built to help protect animals only insofar as it serves humans, this raises concerns about whether 3Rs programs align with the values enshrined in their respective legislation. The prominent role of the 3Rs in current efforts to promote new approach methods might then need to be reconsidered.

This article makes three original contributions: First, it engages directly with the notion of anthropocentrism and sets it apart from other notions, such as disregard for animal sentience, to make it clear what it means to claim that the 3Rs are anthropocentric or that they are not. This helps to put the conflicting views of previous commentators into perspective (Camenzind & Eggel, 2022; Lauwereyns, 2018; Vorstenbosch, 2005) (Section “The 3Rs and the Notion of Anthropocentrism”).

Second, the article illuminates the original philosophical foundations of the 3Rs by drawing on the writings of C. W. Hume (Section “The Original Moral and Strategic Commitments Underpinning the 3Rs”). These writings contain a reasoned and coherent approach to animal ethics that is explicitly non-anthropocentric. However, Hume also strongly committed to a strategic principle of cooperating with vested interests rather than challenging them. This led to an overall program of protecting animals only within the bounds of human interests in using them. While Hume’s writings have received some limited attention (Balls, 2009, 2013; Balls & Parascandola, 2019), the influence of his strategy on the 3Rs has not been discussed before.

Third, the article reflects critically on the lasting merit of Hume’s strategy, arguing that it lacks justification in the context of government policy and governance (Section “The Limited Justification of Hume’s Strategy”). This is because the principle of cooperating with rather than challenging vested interests is prudent only in positions of relative powerlessness (Section “Conclusion”). Overall, this article represents the first attempt to address the question directly as to whether the 3Rs are an anthropocentric framework and what critical lessons can be learned from the answer.

## The 3Rs and the Notion of Anthropocentrism

Anthropocentrism is the view that all and only human beings have intrinsic value, have moral status, or matter morally for their own sake (Fox, 1998; see also Jaworska & Tannenbaum, 2018; Brennan & Lo, 2022). The notions of “intrinsic value,” “moral status” and “mattering” can in turn be understood in terms of moral obligations (Warren, 1997, p. 3; Müller, 2022, p. 32): Anthropocentrism is the view that there are moral obligations to all and only humans, not to any non-human entity.

At its root, then, anthropocentrism is a view concerning the question *towards whom* there exist moral obligations– whom we owe their observation and who is wronged by their violation. This should be distinguished from the question *why* moral obligations exist or *what* they prescribe. As for the “why,” an anthropocentrist can endorse a variety of theories, including but not limited to contractualist (see Carruthers, 2002; Abbey, 2007), Kantian (see Callanan & Allais, 2020; Müller, 2022), and Thomist approaches (see Scott & Coetser, 2015; Macdonald, 2021).

When it comes to the question of *what* moral obligations ask agents to do, an anthropocentrist can advance a variety of views, too. Kant, to name one classic, argued that the treatment of animals affects human capacities that are required for morality, specifically the capacity for sympathy, and so we owe it to *ourselves* to refrain from cruelty to animals (Müller, 2022, p. 60; Regan, 2004, Ch. 5.5). But there is some leeway for theories to disagree on what exactly this anti-cruelty duty demands– for instance, on whether it implies a duty of vegetarianism (see Egonsson, 1997; Denis, 2000; Hay, 2020). This shows that the “to whom,” “why,” and “what” aspects of moral obligation are not completely congruent.

Because anthropocentrism is a view specifically about the “to whom” aspect of obligations, it should not be equated with views about the “why” and “what.” Consider, for example, the view that humans can treat animals whichever way they want, or that the interests of humans should be assigned greater weight than the equivalent interests of animals. Such views are better characterized as “human chauvinism” or “speciesism” (as discussed by Hayward, 1997) or as the rejection of “moral status unitarianism” (see Kagan, 2019).

The difference between the “to whom,” “why,” and “what” aspects of obligations is not always fully appreciated in debates about the 3Rs. For example, Camenzind and Eggel argue that Russell and Burch’s focus on sentient animals is a reason to think that they were not anthropocentrists (2022, p. 497). But this conflates the “what” with the “to whom.” Russell and Burch certainly did acknowledge an obligation to diminish the distress of sentient animals, but this alone does not settle *to whom* agents owe this obligation. It could be an obligation to the animals, to other humans, to a deity, or anything else. Other commentators have argued that Russell and Burch’s focus on diminishing distress shows that they endorsed a specific non-anthropocentric theory, namely, utilitarianism (Schuppli et al., 2004, p. 526; Landi et al., 2015, p. 228; Walker & Eggel, 2020, p. 8). This conflates the “what” with the “why” because a utilitarian grounding of obligations in a value to-be-maximized is not the only option. Russell and Burch could just as well have believed that diminishing distress is a divine command or a demand of a human social contract. In short, when it comes to the question whether the 3Rs are anthropocentric, the mere fact that Russell and Burch endorsed an obligation to reduce animal distress is neither here nor there.

However, some more nuance is necessary here. The “why, what, and to whom” aspects of obligations do not completely align, but they are also not completely disconnected from each other. Assumptions in one domain put certain restrictions on what can be compellingly argued in the other domains. For example, the “why” can restrict the “to whom.” Take the example of Carruthers’s view (Carruthers, 2002, Chap. 5), according to which obligations arise from a social contract in which only rational agents participate. This kind of contractualist grounding of obligations makes it extremely difficult to account for obligations towards animals. The straightforward conclusion is that such obligations do not exist.

Similarly entangled are the “to whom” and the “what.” For instance, if obligations exist only towards humans, then animals can only be protected indirectly by virtue of standing in some relevant relation to humans. This makes it very difficult for anthropocentric theories to explain why we should devote any moral attention to animals who do not stand in that relevant relation to us. For example, a Kantian view that argues that animals matter only insofar as they affect a human’s capacity for sympathy has trouble explaining why humans should care about animals with whose expressions of pain they do not readily sympathize, such as fish, reptiles, or rodents (Müller, 2022, pp. 76–77).

More limitations for anthropocentric approaches arise when they combine direct obligations to humans with indirect obligations regarding animals. The problem is that the former tend to outweigh or restrict the latter. In principle, of course, animal interests do not have any independent weight at all in such theories, as they derive all their apparent weight from overlapping with human interests. For example, an animal’s interest in not suffering matters in Kant’s theory only insofar as it overlaps with the human interest in retaining one’s capacity for sympathy. But the simplest and morally most desirable way to advance the human interests at stake– say, in safeguarding sympathy– typically does not require acting in full accordance with the animals’ interests.

Take the example of the brutalization of slaughterhouse workers. Assume that unrestricted animal cruelty would give workers an increasingly cruel disposition, making them dangerous to other humans. There is a human interest in preventing this effect, protected by fairly uncontroversial obligations of beneficence or nonmaleficence. This overlaps with the animals’ interest in not being made to suffer. But at the same time, some humans have an interest in meat production, and this interest is protected by fairly uncontroversial obligations too– say, to respect the freedom of other human beings. The best solution from an anthropocentric standpoint is to tolerate all the animal suffering that is necessary for meat production and to prohibit only “excessive” violence. This illustrates how anthropocentrism, because it considers animal interests only to the extent that they overlap with human interests, tends to minimize even the indirect moral consideration it grants to animals.

However, it would be incorrect to say that anthropocentrism strictly or “lexically” prioritizes human use interests over animal interests. Lexical priority would be given if interests in exploiting animals always came first and animal interests came after. But animal interests are never considered for their own sake in anthropocentric views at all, not even after all human interests have been attended to. On the other hand, situations can arise in which animal-friendly human interests prevail over animal-harming ones. For example, the practice of holding animal fights may be prohibited because the interest in safeguarding sympathy in this case outweighs the socially marginal interest in enjoying the spectacle. Thus, although anthropocentrism only considers animal interests insofar as they overlap with human interests and those human interests are often heavily restricted by direct obligations to humans, it does not strictly give lexical priority to human interests in using animals over human interests in protecting them.

Scholars who argue that the 3Rs are anthropocentric are concerned with the preference the framework gives to research interests over animal interests. The 3Rs concept is set up in such a way that the research objective is never questioned (Tannenbaum & Bennett, 2015, p. 123). What we are to ask ourselves, according to Russell and Burch (1959), is whether there are any alternative methods, any ways to reduce the sample size, or ways to make the experiment less harmful to the animals, all without compromising scientific quality. We are however not asked to reflect on our choice of research objectives (what objectives are worth pursuing?– see Beauchamp & DeGrazia, 2020, p. 22) or on opportunity costs (should we better pursue another bit of knowledge that does not require harm to animals?– see Lauwereyns, 2018, p. 109). So, in deeds if not in words, the 3Rs do treat research objectives as lexically superior to animal interests.^2^

Vorstenbosch (2005, p. 341) argues that the 3Rs are anthropocentric because they assume that science is justified by its benefits for humans. Animal interests can justify more humane techniques, but they never make it impermissible to pursue a specific research objective. Similarly, Lauwereyns argues that Russell and Burch “suggested that we should just concern ourselves with avoiding ‘inhumanity’ in the technique” without asking whether the research objective itself was morally justified, and that in this way, their view “always, in every single case, places humans above other animals” (2018, p. 14). In sum, the argument of critics who charge Russell and Burch with anthropocentrism is that the 3Rs treat the research objective as sacrosanct, considering animal interests only within the bounds set by the research endeavor.^3^

Of course, one could argue that at the time of Russell and Burch’s project, the Cruelty to Animals Act of 1876 (article 3, sect. 1) allowed experiments only “with a view to the advancement by new discovery of physiological knowledge or of knowledge which will be useful for saving or prolonging life or alleviating suffering.” While a formal requirement for harm-benefit analysis only entered British law with the Animals (Scientific Procedures) Act of 1986, there was thus already some minimal regulation about acceptable research purposes. Equally, however, the Cruelty to Animals Act already contained certain requirements of refinement by prescribing the use of anaesthesia for painful experiments (article 3, sect. 3), and this did not stop Russell and Burch from thinking in greater detail about how refinement can be achieved and further improved. Thus, even though their silence about acceptable research objectives does not imply that Russell and Burch approved of *just any* research goal, one can ask if their unilateral emphasis on method selection rather than question selection is due to an anthropocentric tendency.

Notice, however, that the implicit prioritization of interests in the 3Rs framework is in fact more extreme than in anthropocentrism, as it assigns truly lexical priority to research objectives over animal interests. An anthropocentric approach would still have us question research objectives and minding opportunity costs. This is because these theories focus on *all* human interests, not just on human *research* or *animal use* interests. The 3Rs, by contrast, are built to accept the latter in every case.

Within the limits of the research objective, however, the 3Rs do not discriminate between animal interests that overlap with human interests and those that do not. Russell and Burch were concerned with animal distress (1959, Chap. 2), not with how animal distress affects humans. Their emphasis on the words “humanity” and “inhumanity” can be misleading here at first glance, as it seems to highlight the moral disposition of the human agent. But Russell and Burch made it exceedingly clear that these terms must only be understood “in a purely objective sense to characterize the kind of treatment actually applied to an animal– in terms of the effect on the latter” (ibid.). While, to repeat, the mere fact that Russell and Burch focus on sentient animals or on reducing animal distress does not show that they were not anthropocentrists, the fact that they explicitly focus on animal distress *irrespective of its impact on humans* is indeed suggestive of a non-anthropocentric ethic.

As a preliminary result, we can see that the 3Rs concept as presented by Russell and Burch is beyond anthropocentrism in two apparently contrasting ways: On the one hand, it prioritizes the research objective so strictly that it gives even less consideration to animal interests than anthropocentrism would, apparently accepting any research objective within the confines of United Kingdom legislation at the time. But on the other, it assigns moral significance to animal distress irrespective of any relation to humans, if only within the limits set by a given research objective. The reason for this odd combination, the next section will argue, lies in the philosophy and animal welfare strategy from which the 3Rs originally arose.

## The Original Moral and Strategic Commitments Underpinning the 3Rs

While Russell and Burch did not offer much in terms of ethical argumentation, their client and employer did. This was the Universities Federation for Animal Welfare (UFAW), an academic animal welfare organization, headed by its founder Charles W. Hume. His connection to the 3Rs was very close, as he had initiated the project of writing *The Principles of Humane Experimental Technique*, was involved in hiring decisions, and gave (sometimes rather harsh) feedback on the work in progress (Balls & Parascandola, 2019). Hume himself regularly spoke and wrote about scientific, philosophical, and theological issues, publishing many of his thoughts in independent scientific journals and in UFAW-edited periodicals and books (Hume, 1956, 1962). These works provide a coherent approach to animal welfare ethics and organizational strategy that helps to better understand the intellectual basis of the 3Rs.

First of all, Hume’s philosophy is unambiguously non-anthropocentric. In his *The Status of Animals in the Christian Religion*, Hume wrote: “[…] a Christian’s duty to his neighbours cannot logically be restricted to neighbours belonging to the same species as himself. Charity is indivisible” (1956, p. 73). Hume’s basic argument for this conclusion is negative: There is no good reason to exclude animals from the scope of moral obligations. Human-animal differences are gradual, not categorical, when it comes to sensation, pain, learning, reasoning, language, attention, aesthetics, emotions, personality, and perhaps even the sense of morality (1956, pp. 40–49). Hume reasoned that one cannot categorically exclude animals from moral consideration based on merely gradual differences.

The only categorical distinction Hume was willing to entertain concerned the afterlife and the immortality of the soul. He was skeptical of the default Christian view that denies animals an immortal soul, questioning its theological pedigree on the grounds that it stems from Aristotle rather than scripture: “[…] the doctrine that animals have no souls which can survive death is of pagan, not Christian, origin” (1956, p. 49, similarly 1962, p. 183). The argument that most convinced Hume was that God made human souls immortal in the first place because he loves them and thus wishes to preserve them, an argument which extends to animals too (1962, p. 167, 1956, p. 50). However, for practical purposes, Hume was ready to accept the premise that animals do not have an immortal soul while humans do (see 1962, p. 128), emphasizing that “our duty towards animals is a binding one, whether Aristotle was right or wrong” (1956, p. 50). In sum, Hume made it abundantly clear that he was not an anthropocentrist. To the contrary, he had a deeply-held conviction that we have the very same duties of neighbourly love towards humans and animals alike.^4^

In accordance with his non-anthropocentrism, Hume lambasted the “fanatics who say that mere animals must always be sacrificed to human interests; that any outrage, however horrible, may be perpetrated against an animal if the human race stands to gain by it” (1962, p. 129). Once again, it is abundantly clear that Hume did *not* take human interests in using animals to be lexically superior to animal interests on moral grounds.

Second, Hume’s moral outlook was anti-speciesist *avant la lettre*: “[…] is it more objectionable to hurt a man than an animal? I should say definitely not, and if anybody thinks that it is, it is pertinent to put the question ‘Why?’” (1962, p. 130). On another occasion, too, Hume emphasized that when it comes to the infliction of pain, there is no morally relevant distinction between humans and animals (1956, p. 52).

The implications of Hume’s anti-speciesist view were limited, however, by a more specific argument about the ethics of killing. “To kill an important being is a more serious matter than to kill an unimportant one […]. Human beings are more important than animals, and it is a much more serious thing to kill a man than to kill an animal” (1956, p. 51). By “importance,” Hume meant historical irreplaceability, as his example reveals: “If a cock sparrow is killed by the cat, his hen will in due course find another mate”, but “if the eleven apostles had all been executed on the first Good Friday, there would have been no Christian Church and no Christian civilization. Truly they were of more value than many sparrows” (ibid.). Hume also acknowledged that his view implies that killing people of great historical significance is a greater injustice than killing ordinary folk, but he emphasized again that inflicting pain on both is equally unjust (ibid.).^5^

Although Hume’s argument only implies that killing animals is relatively “less serious” than killing (certain) humans, he went one step further and treated killing animals as morally neutral: “Animals are killed every day in the slaughterhouse (and legitimately, provided they be killed humanely), whereas to kill an innocent human being in peacetime is murder” (1956, pp. 51–52). Hume never delved deeper into the question why killing animals should be morally unobjectionable just because it is painless, but he appeared to assume that animals are simply not harmed by death: “There is no harm in killing animals provided it be done painlessly” (1962, p. 130). While this claim became the object of intense philosophical dispute only considerably later (see Kasperbauer & Sandøe, 2016), Hume also did not engage with opposing views that would have been available to him, such as Henry Salt’s (1894) writings in defense of a right to life for animals.

The strength of Hume’s conviction that animal death is unproblematic deserves emphasis. Apart from painless slaughter, he also endorsed the practice of killing “unwanted puppies and kittens” on the grounds that “only a small percentage of animals can reach maturity in any case, for otherwise there would soon be no room left in the world” (1962, p. 130). Another of his examples involved animal testing: “A method of testing milk for tubercular infection consists in injecting a preparation into guinea-pigs, killing the animals painlessly before the disease has reached a stage where it can cause any suffering, and then making a post-mortem examination. Nobody can reasonably object to that” (ibid.).^6^

Hume’s views had a strong impact on the aims and methods of UFAW. The organization’s stated aim was “to reduce the sum total of pain and fear inflicted on animals by man” (UFAW, 1952), but not to reduce the number of animals killed. In fact, painless killing was a central method of distress reduction for UFAW. The organization published a series of pamphlets on “Kind Killing” that included instructions on how to gas, shoot, or knock dead animals of various species (UFAW, 1950, 1967; see also Nature, 1955). The organization also devoted extensive time and resources to determine the conditions under which electrocution was painless in meat production (Hume, 1962, pp. 71–92), while never recognizably supporting lower meat consumption or collaborating with any vegetarian societies of its day. Overall, UFAW in Hume’s day devoted considerable efforts to the promotion of painless killing as a method of reducing distress. Russell and Burch, too, hail working with painlessly killed animals as a harmless replacement technique (1959, Chap. 5).

However, even if one shares Hume’s moral views, they do not explain why the 3Rs treat research objectives as having lexical priority over animal welfare. We have seen that Hume endorsed a non-anthropocentric and anti-speciesist view that emphatically denied that death is a harm to animals, but such an approach could still advocate that certain research questions should not be investigated simply because they would require great animal suffering, or that one should purposely choose one’s research objectives so that they are achievable without animal distress. To understand why Russell and Burch did not develop a framework that instructs researchers to do this, we must consider not just Hume’s *moral* views but also the *strategic* views he expounded in his writings.

Hume’s strategy rested on two key commitments: The first was that strategies should be determined by facts, not feelings (1962, p. 13). Both sides of the coin– being guided by facts and *not* being guided by feelings– were important for UFAW’s organizational identity. Given its academic background, UFAW laid emphasis on taking a scientific approach to animal welfare issues (Hume, 1962, p. 14). According to Hume, UFAW prioritized issues in proportion to the intensity and duration of suffering involved, the number of animals affected, and the feasibility of practical reforms (1962, p. 15). On the flipside, Hume thought that laypeople are poor judges of what should be done for animals due to ignorance and sentimentality– “the welfare of animals depends on factors lying beyond the ken of many animal-lovers” (1962, p. 14). He thus emphasized that UFAW did *not* choose its policies by popularity and was *not* influenced by “cranky pressure groups” (1962, p. 15). To Hume’s mind, this was the problem of traditional animal welfare organizations who devoted too much of their time to rescuing stray animals and of antivivisectionists who made popular, but unrealistic demands (1962, pp. 13–14). UFAW also ran ads that read “are you interested in animals without being a fanatic?” (UFAW, 1958), emphasizing its anti-sentimental and pragmatic approach.

The second key commitment of Hume’s strategy lay in cooperating with agents who use animals rather than challenging them, such as farmers and scientists. For a start, Hume fundamentally rejected any adversarial approach to political activism, writing that “perhaps the most important rule of all for avoiding quarrels and resentments is this: *never to impute motives*” (16, original emphasis). By this he meant that one should not assume any ill will on the part of animal users. This approach is also clearly reflected in Chap. 2 of the *Principles*, where Russell and Burch emphasize in all-capital letters that their terms “humanity” and “inhumanity” “MUST NOT BE TAKEN TO IMPLY ETHICAL CRITICISM OR EVEN PSYCHOLOGICAL DESCRIPTION OF PERSONS PRACTICING ANY GIVEN PROCEDURE” (1959, Chap. 2, original capitalization).

In principle, however, even if one does not assume any ill will on the part of animal users, one could approach them in a challenging rather than cooperative way. As a rule, Hume and UFAW did not do this. “Most people, in this country at all events, have some sympathy for animals”, Hume wrote (1962, p. 16), continuing: “With this fact in mind, UFAW tries to enlist the help of persons who are actively engaged in occupations which entail a risk of suffering for animals” (ibid.). His operative assumption, it appears, is that most people who use animals would prefer to safeguard animal welfare, other things being equal.

It is noteworthy that this approach was taken not just towards animal researchers, but also rabbit trappers and even whalers (1962, pp. 16–17). For rabbit trappers, Hume and his colleagues advocated using a particular, supposedly less painful trap called the Lewis Humane Snare– a campaign for which Hume later apologized, apparently considering it misguided in hindsight (1962, p. 30). For whalers, UFAW promoted the electric harpoon as a refinement alternative to explosive harpoons, but this innovation never took off (1962, p. 215).

It is this maximum of charity towards, and willingness to cooperate with, animal users that truly explains why the 3Rs treat research objectives as sacrosanct and focus only on promoting more humane ways of achieving them, effectively assigning lexical priority to research interests over animal interests.^7^ When it came to animal research, Hume and UFAW sought to enlist animal researchers as voluntary collaborators, which was possible only if their research objectives were accepted as given.

In conclusion, it is by no means moral anthropocentrism that led Russell and Burch to treat research objectives as sacrosanct, but a general strategic approach of cooperating with animal users rather than challenging them. This strategy led Hume’s non-anthropocentric, anti-speciesist moral outlook to promote a 3Rs framework that disregards animal interests even more than anthropocentrism.

## The Limited Justification of Hume’s Strategy

It is not difficult to see the appeal of Hume’s strategy. It avoids direct conflict with vested interests in using animals by focusing on the scientific investigation of ways in which these interests could be satisfied in more humane ways. However, it is worth reflecting critically on the approach and its assumptions.

First, Hume’s claim that everyone has a baseline of sympathy for animals (1962, p. 16) deserves some critical attention. This claim is crucial because it explains why seeking cooperation with vested interests, including rabbit trappers and whalers, is not futile for an animal welfare organization. The problem is that almost any measure to safeguard animal welfare– be it only to switch from an explosive harpoon to an electric one– involves some transition costs. Personnel may need to be retrained (risking resentment and conflict), procedures redesigned, equipment acquired or rearranged. So Hume’s strategy cannot be justified by appealing to the modest assumption that animal users will prefer the more animal-friendly option if all other conditions are equal, but must assume the bolder claim that sympathy can even motivate them to accept extra costs to some relevant degree. Of course, one can argue, as did Russell and Burch (1959, Ch. 7), that humane techniques come with added benefits for humans, such as better data. But even then, there must be some reason why these benefits were not already sought. Among seasoned veterans of a given animal-using trade, this reason will often not be plain ignorance of better options, but transition costs. It is not enough for a more humane harpoon to be more economical once installed, but it also needs to be affordable, easy enough to install and use, and accepted by the crew. Hume’s strategy rests on the fundamental trust that animal users will accept transition costs due to their sympathy for animals. But this is likely true for some agents in some circumstances, not for others. It was evidently not true for whalers, as Hume himself admits that the electric harpoon failed due to “obstruction by gunners” (1962, p. 215).

Yet, contrary to the evidence, Hume assumed as a matter of principle that anyone is sympathetic enough to accept extra costs for the sake of animal welfare. His reasons for this assumption are not entirely clear, but could be sought in his Christian values or in his national pride, since he primarily ascribed sympathy to British people (ibid.) and repeatedly highlighted that other countries were much more cruel to animals (1962, pp. 49, 63, 92, 123, 126–128, 203–204). Hume even blamed the failure of the electric harpoon on Japanese influence (1962, p. 215). Whatever the reasons for Hume’s blanket assumption of sympathy may have been, they seem questionable from a facts-based standpoint. Sometimes, a force stronger than sympathy may be required to motivate animal users to cooperate, such as economic interests or the threat of legal repercussions.

Secondly, in what conditions is Hume’s strategy advisable? Trying to enlist the voluntary cooperation of vested interests may be prudent if one is in a position of relative powerlessness. When rabbit trappers, whalers, and animal researchers have plenty of resources, far-reaching networks, and the law on their side, they are under no pressure to do as activists ask. This is a basic strategic problem for activists. But rather than attempting to reduce the power differential, say, by building up a more powerful movement for animals or by lowering the public standing of vested interests by launching outrage campaigns, Hume and early UFAW chose to work within the power differential. Their approach can be understood to offer low-cost (but not no-cost) animal welfare solutions, such as the Lewis Humane Snare, the electric harpoon, and humane experimental techniques. The key function of these solutions is to appeal to the conscience of the animal users themselves in order to achieve small-but-tangible benefits for animals.

That Hume’s cooperative approach was conditional on a power differential can be seen from the starkly different way he wrote about cruelty in young boys: “Many boys pass through a sadistic phase in which they destroy birds’ nests, maltreat young birds, inflate frogs, torture hedgehogs, etc. Such cases call for either psychiatric or castigatory treatment but are all too common” (1962, p. 217). It would obviously be absurd to call for a cooperation with young boys to determine scientifically how they might inflate frogs in more humane ways, perhaps by killing the frogs painlessly first. Adults have power over children, so the problem is straightforwardly addressed by mandatory rules and not voluntary cooperation.

Obvious as it is, the conditionality of Hume’s strategic approach on a position of relative powerlessness is important to consider. It suggests that relying on the 3Rs framework, with its hands-off approach to the choice of research objectives, may be more justified in some contexts and less justified in others. It makes some strategic sense for animal welfare organizations because they have no influence over what objectives are being researched. But it does not make the same strategic sense for governments who regulate and fund research. In spite of this, the 3Rs remain central to many governments’ attempts to promote animal welfare in science (see Neuhaus et al., 2022 for an overview).

Of course, in a liberal spirit, governments may want to refrain as far as possible from exercising their power to ban any forms of research. But they also have the power to *fund* some types of research more than others, and to fund research infrastructure, networks, platforms, and conferences according to their own policy goals. They also have the power to create new institutional bodies to oversee and advise on progress. More ideas for how governments can go beyond the 3Rs can be found in political calls for phase-out planning for animal experimentation (see Müller, 2024 for an overview). If a government today recognizes animals as worth protecting for their own sake (as many do, see Kotzmann, 2023), then it should consider policy measures beyond the mere allocation of resources to the 3Rs.

## Conclusion

This article has argued that the 3Rs should not be understood to rest on an anthropocentric moral theory, *pace* previous contributions. A more compelling rationale for the framework’s prioritization of research interests over animal interests is that it rests on an animal-centered ethic that is restricted by strategic considerations in line with the thinking of C. W. Hume. The article closed with critical comments on this strategic approach, highlighting in particular that animal users’ willingness to cooperate should be judged by the evidence, not based on a blanket principle of charity, and that the cooperative approach is advisable mainly in positions of relative powerlessness. This suggests that governments, who hold various forms of power in their jurisdiction, should explore ways to influence the selection of animal-friendly research objectives and not focus solely on the 3Rs and humane experimental technique.

## Footnotes

1 By a “human” research objective, I mean an objective chosen by the human researcher, not necessarily an objective aiming only at benefit for humans. Even if research aims at benefits for animals (be it the very animal under study, some or all of its conspecifics, or other animals), one can ask why Russell and Burch did not articulate a framework for animal-friendly question selection, but rather one for animal-friendly method selection, accepting questions as given. Direct moral consideration of animals could motivate a much more cautious approach to how we try to benefit them, including that we do not easily harm some animals for the sake of other animals.

2 While the research purpose is indeed taken for granted by Russell and Burch, some more recent approaches to the 3Rs consider reformulation of the research question a form of replacement. This is taught, for instance, in a course by the European Union’s Education and Training Platform for Laboratory Animal Science (ETPLAS, 2021). However, reformulation can only reasonably count as a form of replacement if the research objective remains the same (Kramer, 2023), such as finding a safe and effective treatment for some given condition. So even at its most progressive, current 3Rs teaching has departed only to a limited extent from Russell and Burch’s approach that targets method selection but not topic selection.

3 Marino (2020) also criticizes this prioritization but does not use the term “anthropocentrism.”

4 Hume’s explicitly religious argumentation further undermines the claim that the 3Rs are a utilitarian construct at heart (see section “The 3Rs and the Notion of Anthropocentrism”), as this would make it a secular approach. Hume’s ethic is better understood as based on a Christian idea of neighbourly love that extends beyond the species.

5 An implication Hume apparently did not see is that there could be people so historically replaceable that killing them is like killing a sparrow, and that these would make for excellent scientific models of the human body. Thus, even though this was certainly not Hume’s intention, his view might inadvertently imply the permissibility of experimenting on certain painlessly killed humans.

6 Of course, all it would take to reasonably object to that is the view that death is, in fact, a harm to animals.

7 Again, it may be noted that the Cruelty to Animals Act of 1876 contained some conditions for acceptable study purposes, but this does not explain why Russell and Burch did not try to improve on this aspect of the Act. They did, by contrast, try to improve on the Act’s requirement for refinement in the form of anaesthesia. This emphasis is best explained by UFAW’s organizational focus at the time.
